# The determinants of investment fraud: A machine learning and artificial intelligence approach

**DOI:** 10.3389/fdata.2022.961039

**Published:** 2022-10-10

**Authors:** Mark Lokanan

**Affiliations:** Faculty of Management, Royal Roads University, Victoria, BC, Canada

**Keywords:** investment fraud, machine learning, artificial intelligence, self-regulation, regulatory technology

## Abstract

Investment fraud continues to be a severe problem in the Canadian securities industry. This paper aims to employ machine learning algorithms and artificial neural networks (ANN) to predict investment in Canada. Data for this study comes from cases heard by the Investment Industry Regulatory Organization of Canada (IIROC) between June 2008 and December 2019. In total, 406 cases were collected and coded for further analysis. After data cleaning and pre-processing, a total of 385 cases were coded for further analysis. The machine learning algorithms and artificial neural networks were able to predict investment fraud with very good results. In terms of standardized coefficient, the top five features in predicting fraud are offender experience, retired investors, the amount of money lost, the amount of money invested, and the investors' net worth. Machine learning and artificial intelligence have a pivotal role in regulation because they can identify the risks associated with fraud by learning from the data they ingest to survey past practices and come up with the best possible responses to predict fraud. If used correctly, machine learning in the form of regulatory technology can equip regulators with the tools to take corrective actions and make compliance more efficient to safeguard the markets and protect investors from unethical investment advisors.

## Introduction

In recent history, self-regulation in the Canadian securities industry has come under criticism for not regulating in the public interest (Fair Canada, 2014; Lokanan, 2017; Kenmar Associates, 2020). Most of these criticisms centered on the enforcement of complaints by the two self-regulatory organizations (SROs) responsible for policing Canada's securities market and to regulating and protecting investors from fraud victimization—-the Investment Industry Regulatory Organization of Canada (IIROC) and the Mutual Funds Dealer Association of Canada (MFDA) (Langton, 2019; Carson, 2020). Research confirmed that fraud detection is an ongoing problem for regulators and encouraged them to raise their profile and find ways to ensure earlier detection and intervention of investment fraud (Fair Canada, 2014; Canadian Securities Administrators, 2020). When fraud is detected, investment advocates argue that enforcement is weak and that the imposition of penalties by SROs have been inconsistent and not proportionate to the harm caused by the offense (Gray and McFarland, 2013; Fair Canada, 2014; Canadian Securities Administrators, 2020; Lokanan and Liu, 2021).

To address these concerns, the Canadian Securities Administrators (CSA) and the Ontario Securities Commission's (OSC) in 2020 set up the Capital Markets Modernization Taskforce (Taskforce) and released the CSA Consultation Paper 25-402 Consultation on the Self-Regulatory Organization Framework, seeking input from stakeholders to streamline Canada's SROs and address issues related to regulatory inefficiencies and the weak enforcement of complaints (Kivenko, 2020, para. 4). A key part of the consultation is to examine the existing framework of the IIROC and the MFDA to create a single more powerful SRO that would do a better job to identify red flags of fraud before they occur and to ensure that penalties imposed for rule violations are proportionate to the harm caused to investors. In January of 2021, the Taskforce released its final report to the Ontario Minister of Finance, including recommendations to use regulatory technology and computational intelligence to modernize SROs governance and protect the public interest. In November of 2021, the CSA announced a new enhanced SRO initiative in Canada.

Indeed, self-regulation has the imprimatur of a statute in the *Securities Act* of Ontario and British Columbia. Given this renewed interest and support for self-regulation in Canada's financial markets, it is opportune to revisit the effectiveness of SROs to govern investment advisors, and securities dealers from the prohibited transactions rule under the provincial *Securities Acts*. The IIROC is one of Canada's SROs responsible for policing investment dealers and brokerage firms involved in debt and equity trading in Canada's capital markets. Based on the IIROC's role as the self-regulatory oversight organization for investment advisors and dealers trading on Canada's marketplace, in what ways can IIROC promote ethical behavior, protect investors from fraud victimization and regulate in the public interest? This paper aims to employ machine learning algorithms and artificial neural networks (ANN) to predict investment fraud and identify the features that contribute to the financial exploitations of investors in Canada. The objective is to design and develop a fraud classification model that will allow regulators and law enforcement to predict the probability of investment fraud using supervised cost-sensitive machine learning and artificial intelligence (AI) techniques based on investors, offenders, and enforcement attributes as useful criteria to assess the ethics of financial market behavior.

A rich conceptual apparatus and theoretical traditions convey how industry self-regulation should be defined and applied in financial market regulation. The motivations that lead industry actors and associations to invest in self-regulation and why government officials encourage self-regulation by delegating powers to SROs have been well-documented in the literature (see Ayres and Braithwaite, 1992; Baldwin et al., 2011; Ogus and Carbonara, 2012; Lokanan, 2018a). Less studied, however, is the significance of the connection between private agents investing in self-regulation and the occurrence of a particular form of industry self-regulation. One way to construe this connection is to view self-regulation as a defense to justify its existence when the confidence in the market has declined due to the fraudulent activities of agents and financial crises. Besides providing a more nuanced approach to regulatory studies, this paper makes two core contributions to the literature and practical application of self-regulation in capital markets.

Successful self-regulation in Canadian finance is important because government regulation is completely ineffective. Canada is unique in having its “patchwork” system of inept provincial regulators. It is also notable for lax criminal enforcement for crime in the sector (see Brownell, 2015). As such, there is a clear need to understand better the efficacy of SROs in enforcing securities fraud and transgression in financial markets/securities trading in Canada. The examination of self-regulation to safeguard the public interests is influenced by the view that regulation is desirable only when the markets fail to protect the public interests. There is no desire to apply any efficiency theory of regulation, but only to recognize that the designers of the rules and regulations governing Canada's capital market should be concerned about financial exploitations and financial abuse to Canadian investors. In this regard, this paper goes beyond the prescriptions for self-regulation to dive deeper into the financial market manipulation and overarching self-dealing with dealers that violates industry ethics and morality. This paper contributes to a body of literature that examines the purview of SROs to facilitate market realities and the use of regulatory technology to protect the public from financial abuse.

Practically, this study addresses a real-world problem facing SROs' policing functions in Canada. Feedback from the CSA's consultations reveals that much of the argument to streamline the SRO's framework hinges around cost savings for the dealer firms. However, the University of Toronto Faculty of Law Investor Protection Clinic (IPC) and the OSC's Investor Advisory Panel (IAP) both noted that protecting investors and the enforcement of complaints should be considered as equal reasons for SRO reform (Investor Advisory Panel, 2020; Investor Protection Clinic, 2020). The Portfolio Management Association of Canada (PMAC) and the Private Capital Markets Association of Canada (PCMA) echoed the IPC and IAP concerns and noted that the SRO system had been criticized for ineffective regulation, particularly in enforcement and fraud prevention (Portfolio Management Association of Canada, 2020; Private Capital Markets Association of Canada, 2020). This paper takes stock of these concerns to conduct a scientific inquiry into fraud detection and financial abuse in Canada's financial market. It is expected that the findings from this project will inform the SROs' consultation process on securities fraud and transgression in financial markets/securities trading. Fraud detection can be more effective when machine learning, and AI techniques can use historical data to predict the probability of fraud from new entries.

The rest of this paper is structured into four sections. The first presents a review of the literature on self-regulation in finance. The second outlined the research methods and the algorithms used in the model. The third section provides an analysis of the findings. The final section discusses the results and provides a conclusion of the wider application of machine learning and AI for fraud prediction.

## Self-regulation in finance

This paper is anchored with a self-regulatory theoretical framework, using a sociological definition of “self-regulation.” The most common conceptualization of self-regulation involves government delegation of power to a quasi-governmental body tasked with preventing unethical behavior and criminal misconduct by regulating the behaviors of its members (Brockman, 2008, p. 588; Lokanan, 2015, p. 460). The theoretical justification for self-regulation is that it works in the public interest and, in so doing, benefits the industry (Brockman, 2004). Both the regulator and the industry have different objectives and views of self-regulation. From the regulator's point of view, self-regulation is a way to “adopt policies which improve observable features of the activity and give the appearance of service unity” (Ogus and Carbonara, 2011, p. 239). The focus is “directed toward promoting transparency and the ability of market participants to make informed choices” (Engdahl, 2018, p. 580). Unease with the normative asymmetry, regulated industries, on the other hand, view self-regulation as a process that is more within their control to shape the direction of the market and ward off government intervention (Norman, 2011). Self-regulation influences information flow and oversees the marketplace to ensure fair and transparent transactions (Christmann and Taylor, 2006; Heath, 2006; Engdahl, 2018).

Government and private agencies have made investments in SROs that aim to create fair and transparent markets (Heath, 2006; Weismann, 2009; Norman, 2011; Engdahl, 2018). The government, for its part, has delegated power to the SROs to regulate the public interests. To maintain oversight, government officials have acted from a distance and encouraged SROs to enlarge the scope of their work to safeguard the financial markets (Jordana and Levi-Faur, 2004, 2010; Levi-Faur, 2005; Weismann, 2009; Engdahl, 2018). The resultant effects are different forms of regulation: co-regulation, hybrid, state, and pure self-regulation to address problems that government officials are too distanced to address (Engdahl, 2018, p. 570). The absence of direct government regulation means that SROs set and monitor their own rules and enforce violations of those rules. This is not to say that governments have *withdrawn* from policing the financial markets; instead, regulation is in a state of plural policing where the presence of the state is *redrawn* and extended (Crawford, 2006, p. 471). While decentering the rules and enforcement of the rules to SROs may be seen as a withdrawal of the states from financial market governance, their monitoring and policing roles points to the extension of government oversights in the regulation of the financial markets (Crawford, 2006; Norman, 2011; Engdahl and Larsson, 2015).

### Fraud detection using machine learning

Fraudulent activities cost businesses billions of dollars every year. As a result, there is a growing demand for effective fraud detection systems. Machine learning is a promising approach for detecting fraud, as it can learn to identify patterns of behavior indicative of fraud (Lokanan and Sharma, 2022). Supervised machine learning algorithms can be trained on labeled data to classify transactions as either fraudulent or non-fraudulent (Fayzrakhmanov et al., 2018; Botchey et al., 2020). Once trained, these algorithms can be deployed in production to flag suspected fraudulent transactions automatically. Additionally, unsupervised machine learning algorithms can be used to detect unusual patterns of behavior that may be indicative of fraud (Hooda et al., 2018). By applying machine learning techniques to fraud detection, businesses can significantly reduce their financial losses due to fraud.

Research on fraud detection using machine learning is still in its early stages (e.g., Hajek and Henriques, 2017; Hooda et al., 2018; Lokanan and Sharma, 2022). However, the existing evidence suggests that machine learning algorithms may be able to improve the accuracy of fraud detection. For instance, recent research has found that machine learning algorithms can accurately identify fraudulent transactions with very low error rates (Perols, 2011; Omar et al., 2017; Lokanan and Sharma, 2022; van der Heijden, 2013). Furthermore, machine learning can automatically detect fraud patterns that would be difficult to detect through manual detection (Moll and Yigitbasioglu, 2019; van der Heijden, 2013). For example, Huang and his colleagues used machine learning algorithms to detect financial statement fraud with high predictive accuracy (Huang et al., 2014). Similarly, Lokanan and Sharma (2022) and van der Heijden (2013) was also successful in using machine learning classifiers to predict financial fraud.

There are many different types of fraud. As such, it is important to be able to adapt the machine learning algorithm to the specific task at hand. For example, credit card fraud can be detected by looking for unusual transaction data patterns for a particular cardholder (see Yee et al., 2018; Fayzrakhmanov et al., 2018). Similarly, insurance fraud can be detected by looking for patterns in claims data that are not representative of the general population (Wang and Xu, 2018). The challenge with fraud detection is that it is often difficult to obtain enough training data to train a machine learning model (see Botchey et al., 2020; Lokanan and Sharma, 2022). Also, the distribution of fraudulent data may be very different from the distribution of non-fraudulent data. These issues can make it challenging for an algorithm to generalize population parameters from training data.

Nonetheless, research on fraud detection using machine learning is underway, and the findings to date are encouraging. Machine learning is a promising approach for detecting fraud. The evidence suggests that machine learning can improve the accuracy of fraud detection and automatically detect fraud patterns that would be difficult with manual detection. However, more research is needed to explore the full range of potential applications. This paper contributes to this ongoing stream of research by employing machine learning classifiers to predict investment fraud in Canadian finance.

## Modeling methodology

### Data collection

Data for this study came from cases decided by the IIROC hearing panel. The IIROC was formed in June 2008 through a merger of the Investment Dealers Association of Canada and Market Regulation Services. Correspondingly, data were collected between June 2008 and December 2019. In total, 406 cases were collected and coded for further analysis. Instead of randomly sampling a set of cases, the entire population of cases was coded. Coding the whole population of cases was justified for two reasons. First, a sample of the cases would have discarded some instances and led to information loss. There is no way to preserve the information that would have been randomly removed from undersampling the data. In machine learning, the loss of data can make the decision boundary between the minority (no-fraud) and majority (fraud) class harder to learn from and leads to poor generalization of the validation set (Branco et al., 2016). Second, undersampling the data can lead to systematic bias and produce results that are not representative of the overall population (Chawla et al., 2002; Lokanan and Sharma, 2022).

### Dealing with missing values

Missing values can cause problems in machine learning classification tasks because they compromise the performance of the model (Jerez et al., 2010; Lokanan and Sharma, 2022). These issues arise because missing values can introduce bias and impede the model's ability to learn from the data (Jerez et al., 2010). Data may be missing for several reasons, including errors in data collection and problems with preprocessing (Khan and Hoque, 2020). There are a few ways to deal with missing values, but each has its drawbacks. For example, one way to deal with missing values is to impute the data, which means replacing them with a synthetic value. However, this technique can introduce errors into the data set (Lokanan and Sharma, 2022). Another method to deal with missing values is simply removing them from the data set. However, removing data can lead to a smaller dataset and information loss. When using machine learning for classification tasks, dealing with missing values is a challenge that must be carefully thought through.

There are various approaches to dealing with missing values, each with its own constraints. In this dataset, four of the features had missing values. These features include offenders' experience (12.8%), banked-owned firms (4.1%), losses to clients (3.3%), and the amount of funds invested (2.6%). Since all of the numerical features (i.e., offenders' experience, losses to client account, and amount of funds invested) were left-skewed, the median was used to impute the missing values (see Khan and Hoque, 2020). The mode was used to fill in missing values for the categorical feature “banked owned firm.”

### Variables and measurements

#### Independent variables

Table 1 presents the independent variables (IVs) used in the model. The IVs capture all the features related to investors, offenders, and Dealer members to predict investment fraud. Note also that most of the IVs are numeric with different ranges and units of measurement. Variables measured on different scales may not contribute equally and create biased models. These variables were scaled using a standard scale to ensure that all the data were within the same range. Using the standard scaler technique, the numeric variables were normalized to change the value of the data into a standard scale between 0 and 1, meaning that the minimum value will be 0 and the maximum value will be 1 (Ali et al., 2014). The categorical variables were converted to numerical features, with 0 representing absence and 1 representing presence.

**Table 1:** Independent variables and measurements.

**Variables**	**Descriptions**	**Measures**	**Indicators**
Investors	Number of investors per case	Numeric	
Loss	Amount loss	Numeric	
Invested	Amounted invested	Numeric	
Off_exp	Years in industry	Numeric	
Inv_age	Age of investor	Numeric	
Inv_income	Investor's yearly income	Numeric	
Inv_liquid_asset	Investor's liquid asset	Numeric	
Inv_networth	Investor's net worth	Numeric	
Comissions	Commission earned	Numeric	
Bank_owned	Investment arm of bank	Categorical	Bank-owned; Not-bank owned
Firm_type	Type of investment form	Categorical	Retail; Institutional
Off_sex	Offender gender	Categorical	Male; Female
Occupation	Offender occupation	Categorical	Advisor; Manager; Executive
Discip_hist	Offender prior offense	Categorical	Prior offense; No prior offense
Inv_sex	Investor gender	Categorical	Male; Female
Inv_Emp	Employment status of investor	Categorical	Employed; Not-employed
Inv_Retired	Investor's occupational status	Categorical	Retired; Not retired

#### Dependent variable

The dependent variable (*y*) is fraud. Section 380(1) of the *Canadian Criminal Code* has a two-part definition of fraud to mean (1) a prohibited act of “deceit, falsehood or other fraudulent means;” and (2) that the act deprives the public or specific person of “any property, money or valuable security, or any service” (Canadian Criminal Code, 1985, C-46). The prediction problem was modeled on whether fraud was committed or not. The binary variable *y* represents whether fraud will be committed as follows:


$$y\;=\left\{\begin{array}{c} 1,fraud\\ 0,\;no-fraud \end{array}\right\}$$

When *y* = 0, there is no fraud; when *y* =1, there is fraud. As can be seen in equation 1, only 5% of the minority class sample was classified as fraudulent.


(1) $$\begin{array}{l} Fraud_{cases\;}=\frac{Fraud}{n\;observations}\;*100=\frac{21}{385}=0.05 \end{array}$$

For more even distribution, the Synthetic Minority Over-sampling Technique (SMOTe) was used to equalize the sample. SMOTe is a machine learning technique that uses *the k*-nearest neighbor closest to the data points to create synthetic samples for the minority class (in this case, fraud) to evenly match the majority class (non-fraud) samples (Chawla et al., 2002, p. 327). Although SMOTe is an excellent algorithm to balance the data, it can lead to over-generalization (Liu et al., 2021). To address the issue of overgeneralization, a hybrid sampling algorithm combining SMOTe and the Edited Nearest Neighbor (ENN) technique was used for data balancing. The SMOTe+ENN technique works by oversampling the minority class and then editing the resulting dataset so that any samples too close to the boundary between classes are removed (Xu et al., 2020). The resultant effect is a dataset more representative of the true class distribution and less likely to overgeneralize (Lin et al., 2021).

### Algorithm selection

The algorithms selected to analyze this dataset are as follows: *k*-nearest neighbors (KNN), Gradient Boosting Classifier (GBC), Random Forest Classifier (RFC), and ANN. These algorithms were selected because they have built-in features to deal with high-dimensional data and categorical variables. They also have features to handle overfitting problems and minimize the loss function during model training.

#### KNN

One of the most popular machine learning classifiers is the k-nearest neighbor (k-NN) algorithm. The k-NN algorithm is a non-parametric method used for classification and regression tasks. The k-NN algorithm, which is wellknown for being both simple and effective, has been successfully implemented in a wide variety of applications, including image, facial expression, and voice recognition (Chen et al., 2018; Jo et al., 2018; Kumar and Rao, 2019). The k-NN algorithm can be very effective in classification tasks because of its ability to automatically learn complex patterns from the data (Fan et al., 2019). In addition, the k-NN algorithm is relatively robust to overfitting, making it a suitable classifier for tasks where the training data is limited (Jiang et al., 2007).

However, it is important to remember that the k-NN algorithm has a few drawbacks. First, the k-NN algorithm requires a large amount of memory (i.e., computational time) (Jo et al., 2018; Djenouri et al., 2019). When dealing with massive datasets, the lack of memory can pose computational challenges. Second, the k-NN algorithm can be slow when making predictions because a new data point must be compared to all previous training points to determine its distance (Jiang et al., 2007; Chen et al., 2018). Third, the k-NN algorithm can be sensitive to noise in the data and may not perform well on datasets with huge outliers (Djenouri et al., 2019). Despite these limitations, the k-NN algorithm is still a powerful tool that can be applied to many different classification tasks.

The k-NN classifier was chosen for this project because of its ability to deal with numerical and categorical variables. The dataset for this project has lots of variabilities, which makes k-NN a helpful algorithm to predict fraud. Even though k-NN is sensitive to noise in the data, the sensitivity is dependent on the *k*-value (Djenouri et al., 2019). When *k* is set too low, the model becomes too specific and will not generalize well to the data. The model achieves high accuracy on the training data (overfits) but poorly predicts the unseen test data. When *k* is set too high, the model becomes too general and fails to predict the test and train sets (i.e., underfitting) (Jiang et al., 2007). There is no go-to scientific method to find the optimal *k*-value; it depends on the structure of the dataset (Jo et al., 2018). In this case, there are over 350 rows, which are sufficient to have a sufficiently large training set compared to the number of features, thereby reducing potential bias and variance (see Fan et al., 2019). These features make k-NN a useful classification algorithm for this dataset. The formula for the k-NN algorithm is shown in equation 2.

Where:

*N*_0_ represents the *k*-nearest neighbors,

*I*(*yi* = *j*) is the dependent variable that is valued at 1 for fraud and 0 for no-fraud,

(*x*_*i*_ and *yi*) represents class _j_, and

*k*-nearest neighbor *N*_0_ identifies the nearest instances of the class with the largest probability.


(2) $$\begin{array}{l} K\left(Y\;=\;j|X\;=\;x_{i}\right)\;=\;1\sum i\epsilon N_{0}I\left(yi\;=\;j\right) \end{array}$$

#### Random forest

Random forest is a machine learning ensemble algorithm that combines multiple independent decision trees to provide more precise predictions and decrease bias and variance in the model (Fawagreh et al., 2014; Barrett et al., 2020). The algorithm works by constructing a series of decision trees and then combining the predictions of all the trees to make a final prediction. Finally, the algorithm is easily adaptable to new datasets and fraud types, making it a valuable tool for fraud detection.

Random Forest is a good classification algorithm for high-dimensional datasets (Rokach, 2010). Feature bagging makes random forests useful for datasets with large proportions of missing values (Barrett et al., 2020). Random forest is practical because it is resistant to overfitting and is more stable to outliers (Ceriani and Verme, 2012; Schonlau and Zou, 2020). The averaging of many interrelated trees reduces error bias and model variance (Sarica et al., 2017). It is easy to determine the importance of the degrees of influence of the feature variables on the target variable with an RFC (Ceriani and Verme, 2012). Other benefits of RFC are that it is used with data that is not linearly separable, unlike many other classification algorithms (Lokanan and Sharma, 2022). Random forest is relatively easy to use and interpret, making it a good choice for datasets with a large number of features (Ceriani and Verme, 2012).

These characteristics make RFC a popular choice for fraud detection because they can handle many features and resist overfitting (Lokanan and Sharma, 2022). Recent research compared the performance of RFC and other machine learning algorithms for fraud detection and found that random forests had the highest accuracy and the lowest false positive rate (Ceriani and Verme, 2012; Sarica et al., 2017; Lokanan and Sharma, 2022). These studies also showed that the RFC was more effective than other algorithms at detecting rare types of fraud. The findings from these studies show that random forests are a promising tool for fraud detection and could be used more widely in the future.

Despite its benefits, RFC is slower to train than other machine learning classifiers. It is also important to tune the algorithm's hyperparameters to get the best results (Schonlau and Zou, 2020; Lokanan and Sharma, 2022). Despite these disadvantages, random forest is still a powerful and popular machine learning algorithm that can be used for regression and classification tasks. The random forest algorithm is beneficial for this dataset because it works well with continuous and categorical features and is particularly helpful in feature selection (Schonlau and Zou, 2020). The mathematical formula for the random forest model is shown in equation 3.

Where:

*h*_*i*_ is the single-decision-tree,

*y* is the dependent variable, and

*I* represent the independent features.


(3) $$\begin{array}{l} H\left(x\right)=argmax_{Y}\left(\overset{n}{\underset{i=1}{\sum }}I\;\left(hi\left(x\right)=y\right)\right), \end{array}$$

#### Gradient boosting

A member of the ensemble family gradient boosting is a technique where each decision tree is a sequence that tries to correct the prediction errors of the previous tree so that the present tree is always better than the one before (Botchey et al., 2020, p. 8). Gradient boosting trains a set of weak learners and converts them into a single strong learner (Botchey et al., 2020). These predictions are then utilized for training the second weak learner, and so forth. The ultimate strong learner is merely the sum of all weak learners. Gradient boosting is very effective in practice and has even outperformed deep neural networks (Bashir and Ghous, 2020; Aziz et al., 2022; Santos et al., 2021). The algorithm is an effective technique for both regression and classification tasks. In recent years, gradient boosting has been used to develop state-of-the-art models for several tasks, including image classification, object detection, and machine translation (Santos et al., 2021; Ait Hammou et al., 2019; Hammou et al., 2021).

Gradient Boosting has several advantages over other machine learning algorithms. First, it is important to carefully tune the hyperparameters, particularly the learning rate (Hammou et al., 2021). Second, gradient boosting is relatively insensitive to overfitting, meaning that it can be used to train large models with high accuracy (Botchey et al., 2020). Third, gradient boosting is computationally efficient, making it a good choice for large-scale machine learning tasks (Botchey et al., 2020). Consequently, gradient boosting has become one of the most popular machine learning techniques in recent years. It is a powerful machine learning algorithm that can be used to achieve state-of-the-art results on a variety of classification tasks.

However, it is important to tune the hyperparameters carefully and to understand the underlying weak learners (Botchey et al., 2020). Another downside of gradient boosting is that it can be very computationally expensive to train, especially when using a large number of weak learners (Bikmukhametov and Jäschke, 2019). Consequently, gradient boosting may not be the best choice for large-scale datasets (Bashir and Ghous, 2020; Bikmukhametov and Jäschke, 2019). Despite these shortcomings, gradient boosting can be an excellent addition to your machine learning toolkit. Gradient boosting is expected to remain a significant tool for academics and practitioners alike as machine learning evolves.

The main reason for selecting the GBC for this project is that it has several parameters that can be optimized and work well with datasets where minimal effort has been spent on data cleaning, preprocessing and exploratory data analysis. Gradient boosting works well because it builds models intelligently, is highly efficient, and puts more weight on observations that are not easily classified (Botchey et al., 2020). The formula for the GBC is shown in equation 4 below.

Where:

*B (X*_*i*_*)* represents the independent features,

*d (X*_*i*_*)* represents the dependent variable (*Y*) that takes the value of 1if the *i*_*th*_ observations belong to *d* and 0 otherwise, and

logP (*X*_*i*_) predict the dependent variable (*Y*) given *d* number of features.


(4) $$\begin{array}{l} Y\left(y_{i,\;}B\left(X_{i}\right)\right)\;=\;-\;\overset{D}{\underset{d}{\sum }}d\left(X_{i}\right)logP\left(X_{i}\right) \end{array}$$

#### ANN

ANN is a branch of AI that tries to mimic the human brain and find relationships with different datasets (Shahid et al., 2019; Albalawi et al., 2020). The algorithm works by imitating the biological neural networks where connections between simple elements (neurons) are intensified or weakened by an activation function to solve problems (Benkachcha et al., 2015; Hajek and Henriques, 2017; Omar et al., 2017). Compared to other algorithms, ANNs are non-linear models with high flexibility and are suitable for working with different features (Shahid et al., 2019).

Neural networks are well-suited for classification tasks due to their ability to learn complex patterns (Benkachcha et al., 2015). They are also relatively robust to noise and outliers in the data. Due to its proficiency with numeric and categorical features, ANN is the preferred choice for financial fraud applications (Dhamija and Bhalla, 2010; Hajek and Henriques, 2017; Omar et al., 2017). ANN is most efficient when numerical variables are normalized to maintain the general distribution of the data. While ANN slows down training time, it is beneficial to handle complex relationships, making it an excellent algorithm for this dataset (Shahid et al., 2019).

Neural networks can be computationally intensive, requiring a large amount of training data to learn effectively (Omar et al., 2017; Albalawi et al., 2020). In addition, neural networks are often opaque, meaning it can be challenging to understand how they arrive at their results. Despite these limitations, neural networks have shown great promise and are being used in various fields, from finance to medicine (see Abiodun et al., 2018; Tkáč and Verner, 2016).

**Figure 1:**
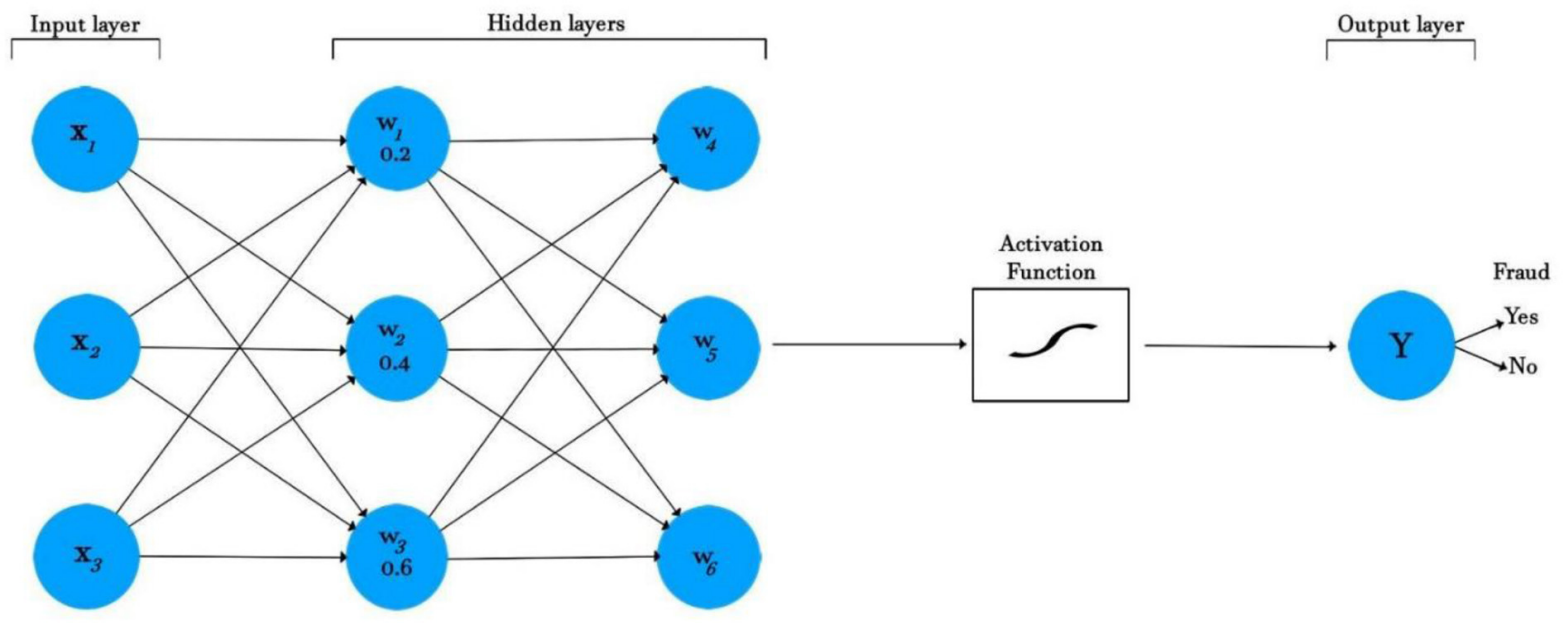
ANN classifier for fraud detection.

Figure 1 shows a diagrammatic illustration of the ANN model. A neural network has three layers: an input layer, a hidden layer, and an output layer. The input layers take the features, process them through an activation function, and then return an output. In Figure 1, three input features are coming into the neural network: *X*_1_, *X*_2_, and *X*_3_, with corresponding weights of 0.2, 0.4, and 0.6. The inputs are then multiplied by the respective weights according to the following formula:


(5) $$\begin{array}{l} Sum\;=\;X_{1}\left(W1\right)\;+\;X_{2}\left(W2\right)\;+\;X_{3}\left(W3\right) \end{array}$$

(6) $$\begin{array}{l} Sum\;=\;X_{1}\left(0.2\right)\;+\;X_{2}\left(0.4\right)\;+\;X_{3}\left(0.6\right) \end{array}$$

The sum is taken, which is offset by the *bias*. The *bias* is a constant (for example, 1), which is added for scaling purposes. The new sum is shown in the formula below:


(7) $$Sum\;=\;X_{1}\left(0.2\right)\;+\;X_{2}\left(0.4\right)+X_{3}(0.6)\;+\;bias$$

The result is then activated to decide the output fraud or no-fraud (range 0, 1).

### Metrics to evaluate a classifier

The decision-making confusion matrix (CM) is convenient for illustrating a model performance. For binary classification, the CM is represented by four possible outcomes:

- **True positive (TP)** - Predict *fraud* when the actual class is *fraud*.
- **False positive** (**FP**) - Predict *fraud* when the actual class is *not-fraud*.
- **True negative (TN)** - Predict *not-fraud* when the actual class is *not-fraud*.
- **False negative** (**FN)** - Predict *not-fraud* when the actual class is *fraud*.

**Figure 2:**
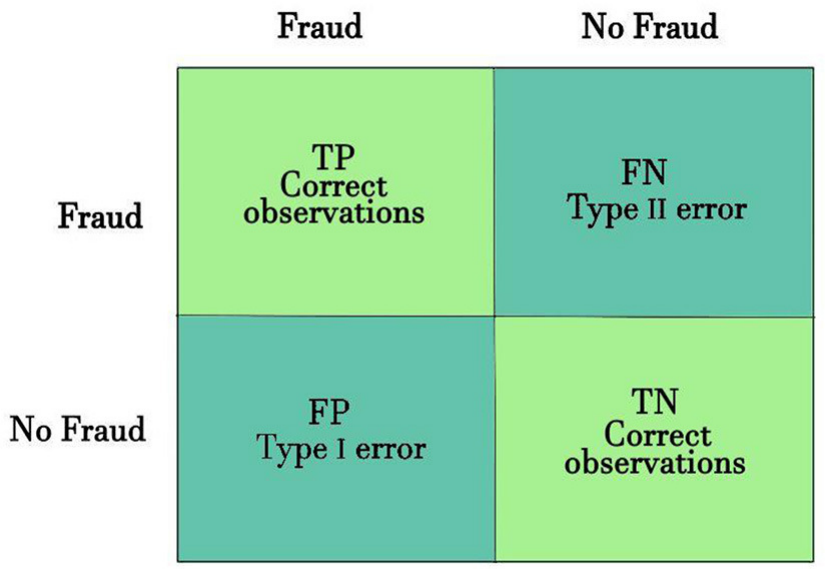
Confusion matrix.

As can be seen in Figure 2, the four outcomes produce two types of true (correct) classification (TP and TN) and two types of false classification (FP and FN).

The accuracy is the most frequently used performance matrix (Hooda et al., 2018). Assuming that the classification model is dealing with balance target classes, the accuracy score on the test set is a good measure of the model performance. However, accuracy is not a good measure of classifier's performance when dealing with imbalanced target classes (Patil et al., 2010; Hooda et al., 2018). The main problem with the raw accuracy score is that it only focuses on the True Positive (Type 1) and False Negative (Type II) errors.

Two other related metrics to the CM are recall and precision (Albalawi et al., 2020; Barrett et al., 2020). The recall is the proportion of all the positive observations (i.e., TP + FN) that are correctly predicted. At the same time, precision is the percentage of the predicted class that the model correctly predicted. The classification algorithm aims to optimize both precision and recall. The F-1 score is the harmonic mean between the precision and recall scores of the positive class (Albalawi et al., 2020).

The Receiver Operator Characteristic (ROC) is another helpful performance metric for imbalanced datasets (Barrett et al., 2020). The ROC curve plots the relationship between the True Positive Rate (TPR) and the False Positive Rate (FPR) at the different thresholds and is a trade-off between TP and FP. The Area Under ROC Curve (AUROC) for a completely random model is 0.5, with a maximum value of 1 (Gao et al., 2019). The closer the ROC curve to 1, the more efficient is the classification model. Table 2 presents the evaluation formula from the CM.

**Table 2:** Classification performance measures.

**Measure**	**Formula**
Accuracy	(TP+TN)/(TP+TN+FN+FP)
Sensitivity, true positive rate, recall	TP/(TP+FN)
Specificity	TN/(TN+FP)
False positive rate	FP/(TN+FP)
Error rate	1-Accuracy

## Findings from summary statistics

Table 3 presents the descriptive statistics of the numerical variables. The average number of investors per case was eight, while the average loss per investor was $365K. Note that the amount of investors lost was in the high range, indicating that investors trusted their advisors with their investment to accelerate their returns (Deliema et al., 2020). The average amount invested was about $2.9 million. The fact that investors' average income was slightly over $12,000 and their liquid asset was about $45,000 corroborates previous research that most of the victims were liquidating fixed assets to try and secure their investment for a better future (Trahan et al., 2005; Lokanan, 2019). Previous studies have used income and net worth as a proxy to measure target attractiveness (Franklin et al., 2012; Leukfeldt and Yar, 2016; Brenner et al., 2020). However, as noted in these findings, income was mostly unobservable to predatory advisors who exploited other features to lure and defraud investors. Note that the investors were not particularly wealthy either, with the losses having a significant adverse effect on their net worth (Brenner et al., 2020). Less experienced brokers (i.e., > 5 years in the industry) are more likely than experienced brokers to defraud investors. The average commission earned from the investment was about $48,000.

**Table 3:** Summary statistics of numeric variables.

**Variables**	**Count**	**Mean**	**std**	**min**	**25%**	**75%**	**max**
Investors	406	8.44E+00	1.22E+01	0	1	10	98
Loss	393	3.66E+05	1.75E+06	0	0	365,823	33,345,000
Invested	396	2.98E+06	8.16E+06	0	246849.25	2,975,602	85,000,000
Commissions	406	4.78E+04	6.17E+04	445	38,992	38,992	688,085
Off_exp	352	1.61E+01	8.62E+00	5	9	23	43
Inv_income	406	1.23E+04	7.87E+04	0	0	0	1,500,000
Inv_liquid_asset	406	4.46E+04	1.93E+05	0	0	0	2,400,000
Inv_networth	406	1.38E+05	1.06E+06	0	0	0	20,000,000

Table 4 presents the summary results of the categorical variables. A common assumption in the fraud victimization literature is that older adults and retirees are more attractive targets to fraudsters because they have more wealth than their younger counterparts and are easier to deceive (Deliema et al., 2020, p. 905). The findings presented in Table 4 show that most victims were unemployed and not retired. These results corroborate the findings from Table 3 that most of the investors were not particularly wealthy. Women are increasingly taking control of their finances and responsible for household financial management (Rutterford and Maltby, 2007; Lusardi, 2012). Therefore, it is not surprising that as more women take control of their household finance, their increased participation in the financial markets will also lead to them being more likely victims of investment fraud (see Lusardi, 2012; Deliema et al., 2020; Lokanan and Liu, 2021).

**Table 4:** Summary statistics of categorical variables.

	**Occupation**	**Bank_owned**	**Firm_type**	**Off_sex**	**Discip_hist**	**Inv_sex**	**Inv_Emp**	**Inv_Retired**
Count	403	389	392	405	326	406	397	394
Unique	3	2	2	2	2	2	2	2
Top	Advisor	No	Retail	Male	No	Female	Unemployed	No
Frequency	316	256	390	361	290	346	364	362

Most of the offenders were investment advisors and belonged to the investment arm of banked-owned firms. Financial intermediaries such as retail investment firms are some of the larger players in the industry. Larger retail firms are usually in unique positions because they often sell complex final products that the average investors cannot understand (Fligstein and Roehrkasse, 2016; Engels et al., 2020). In such cases, investors become vulnerable to the actions of the firms and the investment advisors acting on their behalf. There is an association between male investment advisors and female investors. These findings suggest that male advisors are more likely to gain the trust of female advisors and swindle them in return (see Kadoya et al., 2021; Knüpfer et al., 2021; Lokanan and Liu, 2021).

### Confidence scores of binary classifier

A binary classification of fraud or no fraud may not provide the complete picture of fraudulent activities. Table 5 presents the confidence scores of the balanced accuracy (BAC accuracy), the accuracy range, and their error rates for the binary classifiers at the 95% confidence interval. Note that the classification errors for all the models are relatively low, with the RFC having the lowest error range (0.0009–0.0528). Note also that all classifiers' BAC accuracy and performance accuracy ranges were very high. The low classification errors and the high BAC range and performance accuracy indicate that the models were not prone to misclassifying fraud. While the classification errors are low, there is still a chance that some fraudulent activities could go undetected. As such, it is important to continuously monitor and refine these models to ensure they are as effective as possible at identifying fraud.

**Table 5:** Scores at 95% confidence intervals.

**Algorithm**	**BAC accuracy**	**Accuracy**	**Error range**
	**range**	**range**	
KNN	0.93–0.99	0.93–0.99	0.008–0.071
Gradient boosting	0.92–0.99	0.91–0.98	0.013–0.081
Random forest	0.95–1.0	0.94–0.99	0.0009–0.0528

### Results from machine learning algorithms

#### Performance accuracy

Table 6 presents the performance accuracy of the machine learning algorithms. Note that the base random forest and random forest with GridSearch models have the highest prediction (97%) for the test sets. One of the hallmarks of an overfitted model is a significant gap between the train and test set scores (Linthicum et al., 2019). As shown in Table 6, there are no significant differences between the train and test scores for any algorithm. While the other classifiers' predictions were not as high as the random forest and the GridSearch models, their performance accuracy was not far from the 97% of these two models. Financial institutions do not want to misclassify fraud. Even a small percentage of misclassified instances of fraud can prove fatal. Consider a situation where the financial institution predicted that the individual would not commit fraud but would commit fraud. An FP (type 1 error) can prove fatal and lead to significant losses for the member firm (Fawagreh et al., 2014).

**Table 6:** Performance accuracy.

	**Accuracy Scores**	
**Algorithm**	**Train score**	**Test score**
KNN	0.98	0.96
Gradient boosting	0.1	0.95
Random forest	0.1	0.97
GridSearch	0.99	0.97

When dealing with imbalanced datasets, the raw accuracy score is not a good performance measure. Remember, the main problem with raw accuracy is that it only focuses on true positives and negatives and ignores false positives and negatives (Patil et al., 2010; Hooda et al., 2018). Financial institutions do not want to miss catching fraud (FN). Even though the random forest and GridSearch models performed better than the other algorithms, the error rate was still 0.03, meaning that 3% of the instances classified as fraud were not detected. For these reasons, it is crucial to obtain high precision and recall scores for the models (Fayzrakhmanov et al., 2018).

#### Precision, recall and F1-scores

Table 7 presents the classification reports for each model. The precision for all the classifiers was relatively high, with both the random forest (99%) and random forest, with GridSearch (99%) models being the highest classifiers. These findings indicate that the machine learning models did an excellent job classifying and predicting all the actual fraud observations (Lokanan and Sharma, 2022). High precision scores mean that the results were relevant to limiting irrelevant fraud classification. Notably, the recall scores for all models were extremely high, with the random forest GridSearch model achieving the highest score (99%). Essentially, the models were able to catch most of the fraud cases (i.e., recall) while keeping the cost of detecting these cases under control (i.e., precision) (Fayzrakhmanov et al., 2018). The f1-score further corroborates these results. A high f1-score is critical to identifying every single instance of fraud. It is also important to consider that accuracy lost (FP) translates to monetary loss for the investors and the firms because they must investigate and compensate the victims. In the end, the random forest and the GridSearch models will optimize performance because both classifiers provide the best trade-off between precision and recall scores (Lokanan and Sharma, 2022).

**Table 7:** Scores of performance metrics.

**Algorithm**	**Precision**	**Recall**	**F1-score**
KNN	0.95	0.97	0.96
Gradient boosting	0.95	0.96	0.95
Random forest	0.99	0.96	0.97
GridSearch	0.99	0.99	0.99

#### ROC curve

**Figure 3:**
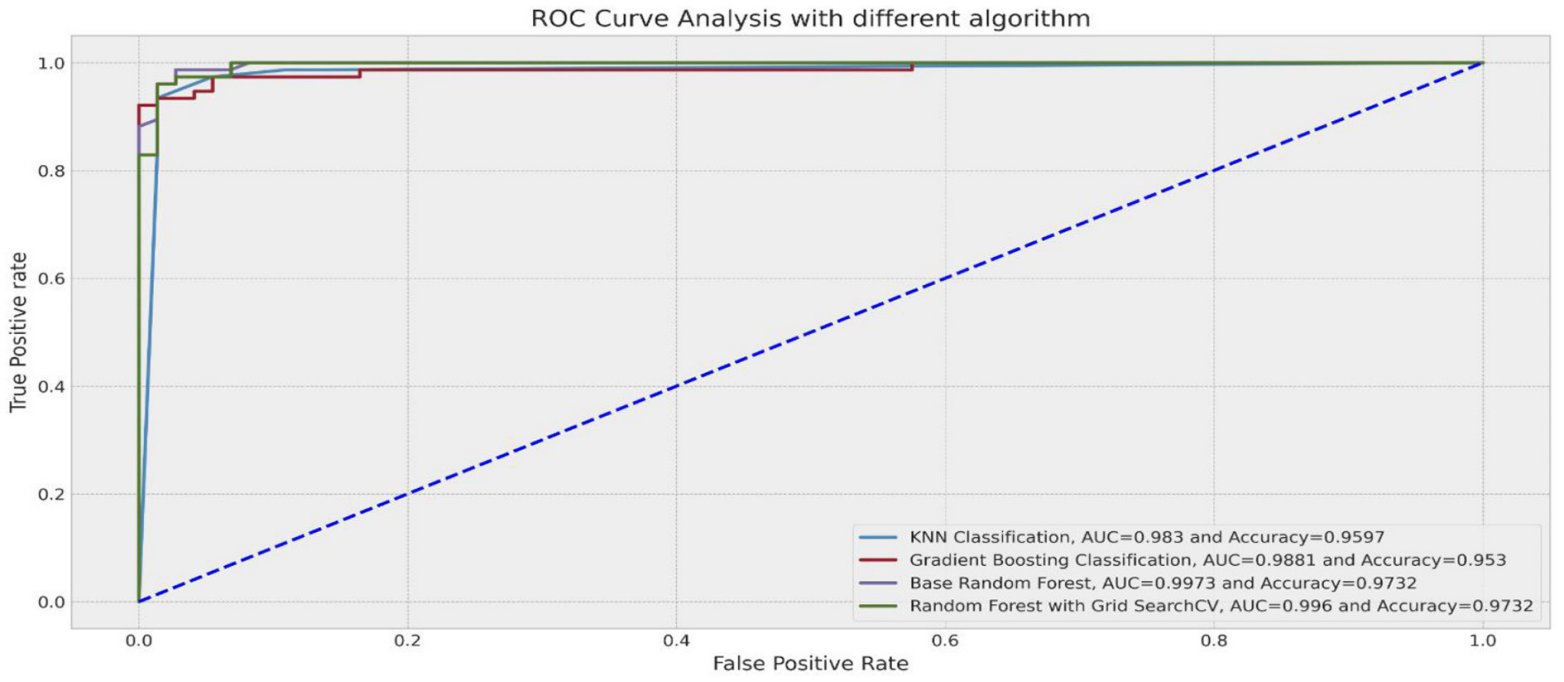
ROC curve.

A more robust measure to deal with imbalanced data is the ROC curve. The ROC curve plots the classification test's sensitivity versus (1-specificity) (Barrett et al., 2020). Recall that sensitivity is the percentage of positive observations correctly predicted as positive, and specificity refers to the proportion of negative observations that were correctly predicted as negative (Barrett et al., 2020). In this case, the Area Under the ROC Curve (AUROC) would have been a more reliable metric to measure performance because it is robust to class imbalance and presents a single quantitative score that considers the class imbalance problem (Fayzrakhmanov et al., 2018; Lokanan and Sharma, 2022). A ROC curve closer to the left indicates a more useful test (Gao et al., 2019; Barrett et al., 2020). Figure 3 presents the results of the ROC curve. The RFC (99.7%) and the random forest with GridSearch (99.6%) were the best performers in predicting fraud because the TPR and the FPR were both closer to the left (and 1) for both models. Note that for the RFC and the GridSeach models, the high ROC score corresponds to the high precision scores shown in Table 7. The ROC score for the RFC and GridSearch models indicates that the number of correctly classified fraud observations was very low compared to the number of incorrectly classified non-fraud observations. These findings demonstrate that the random forest and GridSearch classifiers provide better chances to identify fraudulent observations and avoid misclassifying non-fraudulent observations.

### Neural network learning results

**Figure 4:**
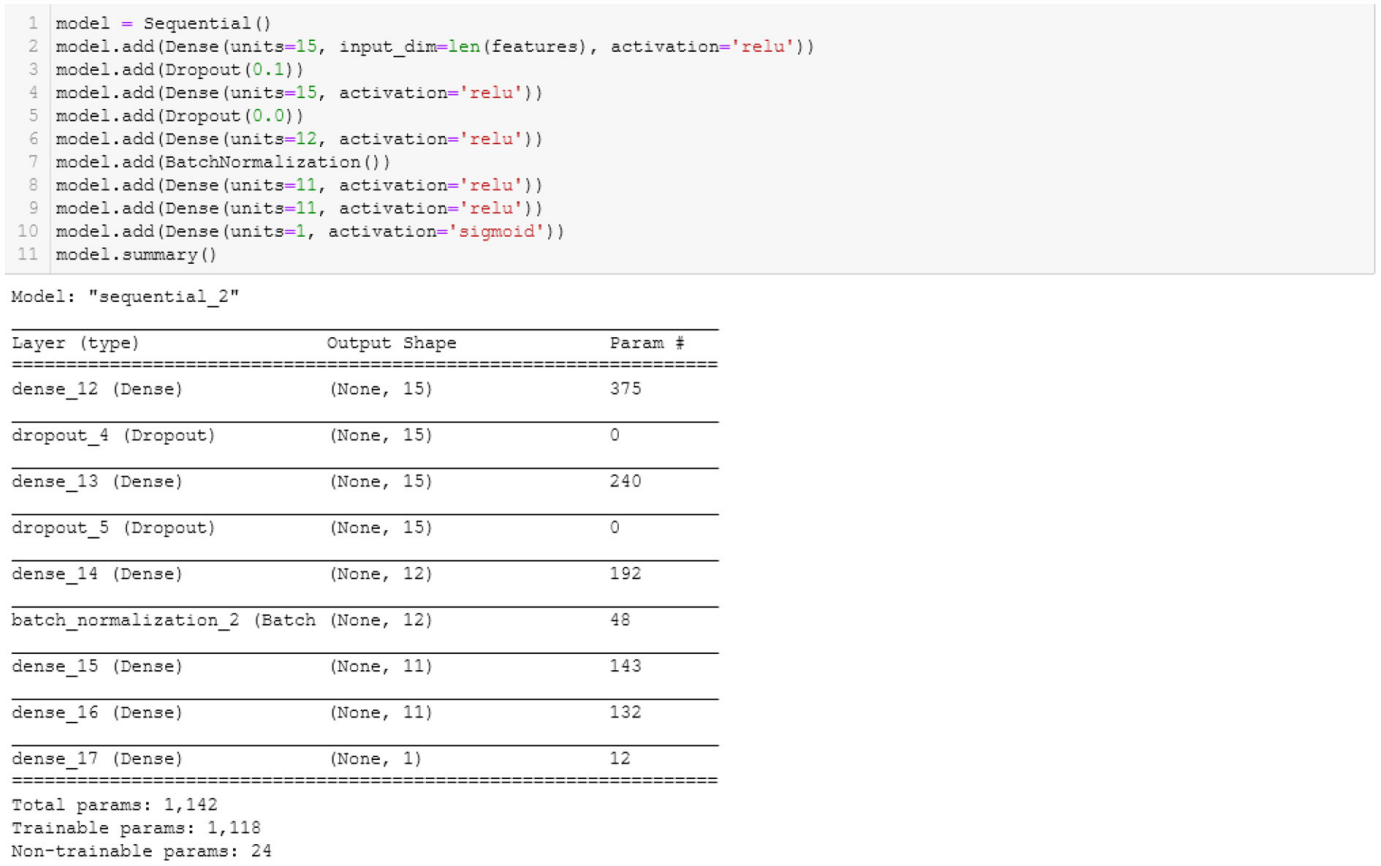
ANN model.

Figure 4 shows the parameters used to build the ANN model. There are six hidden layers. A dropout layer was added to prevent the model from overfitting and ensure that the model learns from the fractions of the weights. Dropout is a useful regularization technique to reduce model overfitting and improve generalization errors in ANN (Srivastava et al., 2014). Batch normalization was included to normalize the inputs from a previous layer before feeding them to the next hidden layer in mini-batches rather than one single input (Keskar et al., 2016). The binary cross-entropy was used as the loss function, and the default optimizer, ‘Adam.' Fifty epochs and a batch size of 200 were the parameters used to build the ANN model.

As shown in Table 8, the performance accuracy of the test set (0.81) was slightly lower than the performance accuracy of the training set (0.86). These findings indicate no signs of overfitting because there is only a 0.5% difference between the accuracy of both the train and the test set. One plausible reason for this outcome is that the dropout layers prevented the overfitting of the model (see Srivastava et al., 2014). Note, however, that the precision score (0.76) decreased significantly from the precision scores of the machine learning models. Although slightly lower than the recall scores of the machine learning models, the ANN's recall score was 0.95, which indicates that the model did a decent job classifying fraudulent observations.

**Table 8:** ANN performance metric.

	**Classification report**			**Accuracy scores**	
**Algorithm**	**Precision**	**Recall**	**F1-score**	**Train score**	**Test score**
Artificial neural network	0.76	0.95	0.86	0.86	0.81

### Feature importance

**Figure 5:**
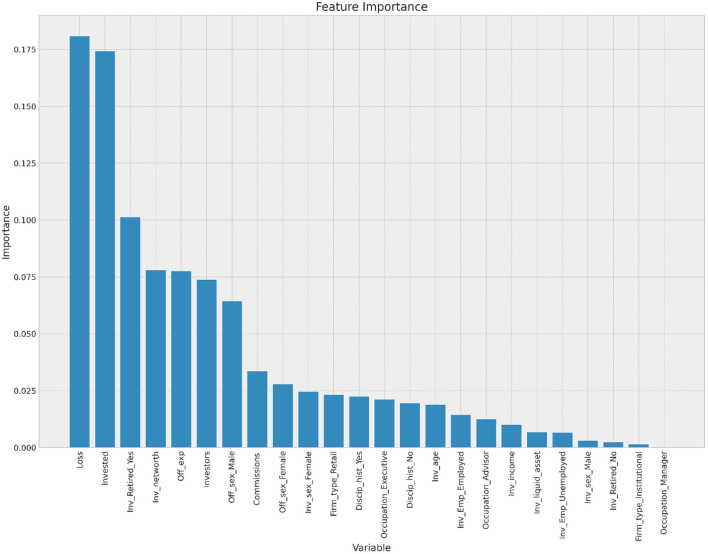
Variables and importance in fraud prediction.

Figure 5 presents the importance of the feature variables for predicting fraud. In terms of standardized coefficient, the top five features in predicting fraud are the amount of money lost, the amount invested, the investor's retirement status, the investors' net worth, and the offenders' experience. These results are important for investment firms and regulators because they help them come up with ways to stop fraud and keep investors safe from possible scammers (Lokanan and Liu, 2021).

The association between the years an advisor spends in the industry and fraud cannot be taken lightly. Anecdotal evidence posits that younger advisors are susceptible to fraudulent conduct because of the pressure to meet quotas and sales targets (Kessler and Hirsch, 2021; Morris et al., 2021). Younger advisers are at a disadvantage when attracting new clients because they lack the network and experience of managing extensive portfolios to convince prospective clients to work with them. The resultant effect is that younger advisors are left to serve existing clients and prioritize risky investments to grow their portfolios (Lokanan and Liu, 2021). The findings presented here provide empirical support that investment firms must take the number of years spent in the industry as a risk factor for future offenses.

The increase in offending risks directly relates to more vulnerable investors falling prey to fraudsters (Lokanan and Liu, 2021) and the amount of funds that are swindled from their accounts (Lusardi, 2012; DeLiema et al., 2016). As noted above, fraud victims lost significant money—-an average of nearly $365K per investor. One explanation is that retired investors are more likely to be swindled of large amounts of funds from their investment accounts than younger investors, either because they are at the peak of asset accumulation or because the fraudsters assume cognitive impairment and retirement make them more vulnerable to financial crimes (Lee and Soberon-Ferrer, 1997; Holtfreter, 2014; DeLiema, 2018). Others argue that older investors may downplay their cognitive impairment and financial independence, making them easy targets for opportunistic financial advisors to win their trust and pilfer their assets (Triebel and Marson, 2012; Holtfreter et al., 2014). The results also show that advisors look for people who seem to have a lot of money and try to get them to invest long-term with their firms, which leads to a more significant overall loss of investments (see McCaffrey and Hart, 1998; Lokanan, 2019).

## Discussion and conclusion

Investment fraud continues to be a problem in the Canadian securities industry (Brownell, 2015; Lokanan, 2018b). It could be that the Canadian SROs are not diligent enough to spot fraud cases or are not conducting enough due diligence on Dealer members to detect and prevent fraud cases before they occur (Lokanan and Sharma, 2022). The manual process of regulators and enforcement officers identifying the red flags of fraud based on their professional judgement leads to inconsistencies in outcomes (Norman, 2011; Lokanan, 2019). In the manual process, enforcement officers focus on the material facts of the cases and provide binary recommendations (fraud or no-fraud) (see Hajek and Henriques, 2017). The machine learning process is based on deep learning predictive rules that identify features from cases that can be used to inform fraud prediction. In doing so, this study adds consistency to the fraud prediction process and provides regulators and Dealer members with a novel approach based on objective science to examine the red flags of fraud before it occurs.

Government can operate efficiently only by proscription, which is seen as drastic in financial market regulations. This approach leaves areas of misconduct and activities that government officials will not touch, some of which are beyond the periphery of the laws and into the realms of ethics and morality (Weismann, 2009). With machine learning, self-regulation can reach into remote areas of conduct because people in the industry are closer to the action and can adopt a more flexible approach to settle disputes that transcends into the realm of ethics and morality (DeMarzo et al., 2005; Christmann and Taylor, 2006; Engdahl and Larsson, 2015). Industry professionals are seen as “the place managers” of the financial markets to act as guardians and safeguard the public interest Engdahl and Larsson, 2015; Lokanan and Liu, 2021. They are assigned the responsibility to police market players and protect vulnerable investors from being targets of investment fraud. In this regard, machine learning in self-regulation helps to improve regulatory performance while retaining human agency in fraud prediction.

Complete due process in financial market regulation is supposed to avoid competition and market abuse so that firms can constrain themselves from pursuing profitable opportunities that are unethical and irresponsible (Norman, 2011, p. 47). The larger industry members, most of which constitute a homogeneous and elite group with identical self-interests, can dominate SROs to create a competitive advantage in the marketplace, which spills over into favorable regulation and sanctions for rule violations (Norman, 2011; Lokanan and Sharma, 2022). Self-regulation, therefore, tends to insulate industry groups and their members from more effective regulation through an illusionary façade of protection that is at the detriment to the public interests.

These perils are exacerbated by comingling the enforcement and regulatory functions of the SROs. Machine learning and AI have a pivotal role in regulation because they can identify the risks associated with these associations by learning from the data it ingests to survey past practices and come up with the best possible responses for the given situation. The algorithm does not care about the association between industry players and regulatory agents; what matters is that the algorithm cares about predicting fraud using historical data. Fraud is a latent variable and very difficult to detect with the current logic-based approach. Machine learning can signal impending risks on the horizon, which can serve as an early warning for regulators to take corrective actions to make compliance more efficient in safeguarding the markets and protect investors from unethical investment advisors.

### Limitations and future work

The model is limited only to the data that reached enforcement. The data used in the analysis were from the cases heard by a hearing panel. Therefore, the predictive model only considers the actual cases that went to a hearing and not all the cases sent to enforcement. In this regard, the algorithms try to predict enforcement rulings, but not all fraud cases reported to IIROC - this could be an important difference for practitioners. That said, enforcement-only deals with the more serious fraud cases and not minor ones. IIROC cannot physically go after all the minor fraud cases and only expend resources on cases where it can secure a prosecution. The focus on the more serious cases is consistent with other fraud detection research (Farber, 2005; Johnson et al., 2009). Increasing the number of fraud cases would have led to the inclusion of less serious cases, which will dilute the sample. More cases would have increased the power of analysis but at the expense of cases that do not technically suit the definition of fraud under the CCC. Therefore, the final sample consists of the more serious fraud cases that are more technically represented as investment fraud.

An essential component of self-regulation is that internal and external oversight must be credible because it is premised on self-policing with regulatory oversight (Weismann, 2009). A corollary of credible internal and external oversight is to have machine learning models in place that could alert staff about possible violative misconduct. Machine learning and artificial intelligence present promising solutions to the uncertainties related to regulatory enforcement by enabling SROs to make optimal use of enforcement data and provide more robustness to the fraud prediction process.

Improvement in computational technology has made it possible to analyze the effectiveness of self-regulation in finance to the degree that would previously have been impossible. New techniques from machine learning and neural networks can be used in financial market research to examine how regulators can leverage these algorithms to detect and prevent fraud. While other machine learning methods can use algorithms to identify abnormal disclosures and the risks of misconduct in corporate documents, data from case files can be examined to identify the possible features of fraud or market misconduct. Regulators and Dealer members interested in fraud detection can employ machine learning algorithms on new data to predict the current likelihood of fraud based on the insights learned from past data. More granular analysis can be done to predict the idiosyncratic risk posed by each investment advisor in a particular account.

## Data availability statement

The data that support the findings of this study is available in Borealis: The Canadian Dataverse Repository at https://doi.org/10.5683/SP3/Q3K08Q.

## Author contributions

The author confirms being the sole contributor of this work and has approved it for publication.

## Funding

This study was funded by the Social Science and Humanities Research Council of Canada. The grant number is 200775.

## Conflict of interest

The author declares that the research was conducted in the absence of any commercial or financial relationships that could be construed as a potential conflict of interest.

## Publisher's note

All claims expressed in this article are solely those of the authors and do not necessarily represent those of their affiliated organizations, or those of the publisher, the editors and the reviewers. Any product that may be evaluated in this article, or claim that may be made by its manufacturer, is not guaranteed or endorsed by the publisher.
